# GSK-3 Inhibitor Promotes Neuronal Cell Regeneration and Functional Recovery in a Rat Model of Spinal Cord Injury

**DOI:** 10.1155/2019/9628065

**Published:** 2019-08-04

**Authors:** Fei Lei, Wen He, Xinggui Tian, Qingzhong Zhou, Lipeng Zheng, Jianping Kang, Yueming Song, Daxiong Feng

**Affiliations:** ^1^Department of Orthopedics Surgery, West China Hospital, Sichuan University, No. 37 Guoxue St., Wuhou District, Chengdu 610041, Sichuan, China; ^2^Department of Spine Surgery, The Affiliated Hospital of Southwest Medical University, No. 25 Taiping St., Luzhou 646000, Sichuan, China; ^3^Department of Library, Southwest Medical University, No. 1 Xianglin Road, Longma District, Luzhou 646000, Sichuan, China

## Abstract

The reparative process following spinal cord injury (SCI) is extremely complicated. Cells in the microenvironment express multiple inhibitory factors that affect axonal regeneration over a prolonged period of time. The axon growth inhibitory factor glycogen synthase kinase-3 (GSK-3) is an important factor during these processes. TDZD-8 (4-benzyl-2-methyl-1,2,4-thiadiazolidine-3,5-dione) is the most effective and specific non-ATP-competitive inhibitor of GSK-3. Here, we show that administering TDZD-8 after SCI was associated with significantly inhibited neuronal apoptosis, upregulated GAP-43 expression, increased density of cortical spinal tract fibers around areas of injury, and increased Basso, Beattie, and Bresnahan (BBB) scores in the lower limbs. These findings support the notion that GSK-3 inhibitors promote neuronal cell regeneration and lower limb functional recovery.

## 1. Introduction

Spinal cord injury (SCI) is caused by trauma and often results in dysfunctional sensations, movement, and permanent loss of sphincter function below the injury level. Its high morbidity is often associated with serious complications that carry a heavy burden for families and society [1]. Repairing SCI is a very complicated process. Studies [2–4] have shown that, in SCI, the microenvironment expresses multiple inhibitory factors that affect axonal regeneration in a prolonged manner. GSK-3 is a highly conservative multifunctional serine/threonine kinase and one of the rate-limiting enzymes in the synthesis of glycogen kinases. It has *α* and *β* isomers, which are homologous. Glycogen synthase kinase-3*α* (GSK-3*α*) and -3*β*, especially GSK-3*β*, are important axon growth inhibitory factors during these processes [5]. Zhou et al. [6] found that changing the dynamics of microtubule growth cones affected axon growth via different signaling pathways, including at the ninth site of GSK-3, to induce serine dephosphorylation and cause its inactivation by regulating factors downstream of microtubule-associated proteins to induce instability. The ninth site of GSK-3 can be serine dephosphorylated to activate a variety of protein substrates and produce a signal transduction cascade that plays a pivotal role in the cell signaling pathways [6]. A GSK-3 inhibitor that acts at the ninth serine dephosphorylation site was combined with factors that induced the ATP-competitive inhibition of GSK-3 activity and consequently promoted axon growth. Jin et al. [7] used mouse neurons in tissue sections and found that appropriately inhibiting GSK-3*β* in a manner selective for its phosphorylated substrates (e.g., CAMP-2 or APC) induced axon branching and promoted axon growth. Dill et al. [8] subcutaneously injected the GSK-3*β* inhibitor lithium (3 mEq/kg/d) and SB (1 mg/kg/d) into an SCI rat model and observed biotin and dextran amine (BDA) expression around the injured area in addition to many corticospinal tract fibers in the cranial and caudal regions relative to the injured segment of the ventral spinal cord.

In this study, we investigated the inhibitory environment in SCI to promote our understanding of the function of the GSK-3 signaling pathway. We used completely paralyzed Sprague Dawley (SD) rats as an experimental model. TDZD-8 was injected into the subarachnoid space of rats. The aim of these in vivo experiments was to observe the effect of GSK-3 inhibition on neuronal cell regeneration and lower limb functional recovery.

## 2. Materials and Methods

### 2.1. Animals

All animal experiments were performed strictly in accordance with the guidelines of the Affiliated Hospital of Southwest Medical University Animal Care and Use Committee. The rats were anesthetized via an intraperitoneal injection of 2% sodium pentobarbital (0.25 mL/100 g or ~0.3 mL/100 g) [9] and injected intramuscularly with cefazolin sodium (5 mg/100 g) according to the WD method [10]. A direct 10 g impact to the T9 vertebra from a height of 25 mm was used to cause paralysis. We developed a homemade Allen hitting system for these experiments. We then exposed the dura at the L4 level and used a l mL syringe to create a hole in the dura. A catheter was inserted approximately 3 mm into the subarachnoid layer (the needle diameter was approximately 0.15 mm), and a catheter was then fixed in place using the paravertebral soft tissue and skin. The model animals were randomly divided into three groups (n=36): a TDZD-8 group (1 mg/kg/d), a PBS group (60 *μ*L/d phosphate buffer solution), and a blank group. All agents were injected at 1 h after SCI, and the rats were observed for 3 weeks. The GAP-43 expression level was measured using SP immunohistochemical staining on days 7, 14, and 28 after injection. The expression levels of markers of neuronal apoptosis were observed using TUNEL staining at 2 h, 4 h, 8 h, 24 h, and 7 d after injection. The motor function of the hind limbs was evaluated in each group using Basso-Beattie-Bresnahan (BBB) scores at 1 w, 2 w, 3 w, 6 w, 8 w, and 12 w after injection. Axonal regeneration was evaluated using immunofluorescence staining at 8 w after injection. Three rats died because of excessive anesthetic, and five rats died of a postoperative urinary system infection.

### 2.2. Neuron Apoptosis

We acquired 20 paraffin-embedded sections from each group at 2 h, 4 h, 8 h, 24 h, and 7 d after injection. These were repaired using a microwave and then closed using proteinase K (1:200, Zsgb, China). dUTP (45 *μ*L/slide) and TdT (5 *μ*L/slide) (1:50 Roche, Switzerland) were added, followed by a second anti-digoxigenin antibody (50 *μ*L/piece) (1:100, Roche, Switzerland) and, finally, DAB (1:20, Boster, China). We then randomly selected five sections in each group and used an Image-Pro Plus system to calculate the average number of apoptotic cells.

### 2.3. Western Blot

Spinal cord tissue samples were stored in a refrigerator at −80°C. They were then removed and cut into 3 mm × 3 mm pieces, homogenized, and joined using liquid nitrogen. Total protein was extracted using a centrifuge. The bicinchoninic acid (BCA) method was used to determine protein concentrations, and the proteins obtained from the tissues were mixed 5 times in sample buffer mix at a ratio of 4:1 and then boiled for 5 to 10 min to induce degeneration. According to the measured protein molecular weights, the samples were prepared and separated on 12% and 5% conventional SDS-PAGE (30 *μ*g/well). The electrophoresis gels were cut, transferred to nitrocellulose membranes, and soaked in 5% skimmed milk powder at 37°C for 1 h. The blocked membranes were then incubated with SIRT6 and GAPDH antibodies at dilution ratios of 1:1000 and 1:1000, respectively, and at 4°C overnight. The membranes were rinsed in room temperature TBST (0.05 mol/L Tris, 0.15 mol/L NaCl, and 0.05% Tween 20) three times for 10 min each time. They were then incubated with horseradish peroxidase (HRP)-labeled secondary antibodies at room temperature for 1 h and washed in TBST three times for 10 min each. The results were visualized using electrochemiluminescence (ECL) chemiluminescence drops.

### 2.4. GAP-43 Expression

A total of 12 paraffin-embedded sections were acquired from each group and placed in a microwave at 7 d, 14 d, and 28 d after injection. GAP-43 expression was observed after the primary antibody (rabbit anti-rat, 1:100, Epitomics, USA) and secondary antibody (mice IgG, Zsgb, Beijing), HRP-labeled chain mildew avidin III antibody (1:200, Zsgb, Beijing) and DAB (1:20, Wuhan Boster) were added. A total of 5 fields were randomly selected for analysis using an Image-Pro Plus system to measure the mean size of the GAP-43-positive area. We calculated the average area of the GAP-43-positive tissues in each section.

### 2.5. Immunofluorescent Traces

The model rats were used to observe immunofluorescent traces at 6 weeks after SCI. The rats were deeply anesthetized and placed in a Benchmark brain stereotaxic instrument system [8]. A hole was drilled near the anterior fontanelle (1.0 mm before or 2.0 mm behind) and at 1.5 mm left or 2.0 mm right of the anterior fontanelle. FITC (fluorescein isothiocyanate) fluorescence tracer was injected into the sensorimotor cortex using a minipump at different depths of the cortex surface (i.e., 0.5, 1.0, or 2.0 mm deep). Each point and depth were injected once with 10 *μ*L. The total volume of FITC administered was 180 *μ*L. At 2 weeks after FITC was injected, the rats were killed to remove the spinal cord, including the trauma region (T9) at its center and extending cranially and caudally. We removed a section of the spinal cord that was approximately 4 cm long. We then used a Leica thermostatic frozen mechanism to cut serial sections with a thickness of 25 *μ*m. We used a CKX41 inverted fluorescence microscope to observe the progress of nerve fiber regeneration in the trauma area. In each section, we randomly selected five regions. These were analyzed using Image-Pro Plus to calculate the average area of nerve fiber regeneration.

### 2.6. Motor Function in the Lower Limbs

The model rats' motor function was assessed at 1, 2, 3, 6, 8, and 12 weeks after injury. Recovery from motor disturbance was graded using a modified murine BBB [10] lower limb locomotor rating scale.

### 2.7. Statistical Analysis

The SPSS 21.0 statistical software package was used. All values are expressed as the mean ±SD. Multiple mean overall comparisons were analyzed using single factor analysis of variance, and comparisons of two groups were analyzed using the SNK-q test.* P* values < 0.05 were considered significant.

## 3. Results

### 3.1. Expression of Neuronal Apoptosis Cells

**Figure 1:**
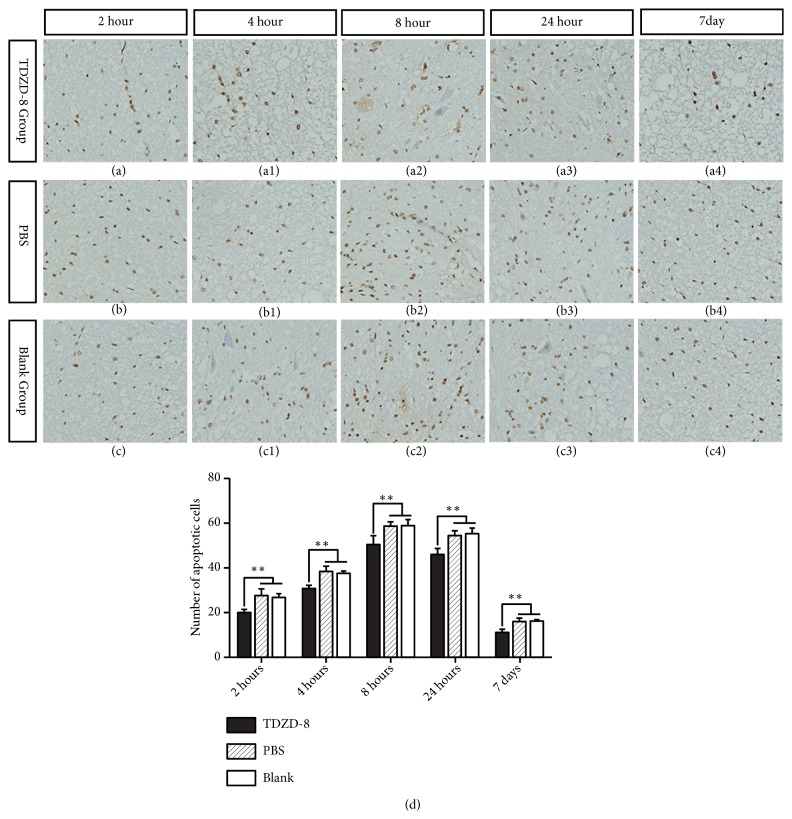
TUNEL staining in rat spinal cord tissue sections. Apoptotic cells are shown at different time points. The number of apoptotic cells gradually increased and reached a peak at 8 h after injury in each group. In the PBS group and the blank group, many apoptotic cells were observed, while few apoptotic cells were observed in the TDZD-8-treated group. *∗P<*0.05 the TDZD-8 group compared with the PBS and blank groups.

Cells positive for apoptosis were primarily distributed in the damaged area and could be visualized at 2 h after injury. Apoptotic cells gradually increased in number and spread to the damage edge at 4 h after injury. Apoptotic cells were also observed in gray and white matter, presenting different morphological types of cells. Apoptotic cells peaked at 8 h after injury and then gradually decreased. At each time point, the number of apoptotic cells in the TDZD-8 group was significantly lower than those in the PBS group and blank group (P < 0.05, Figure 1).

### 3.2. Expression of GAP-43

**Figure 2:**
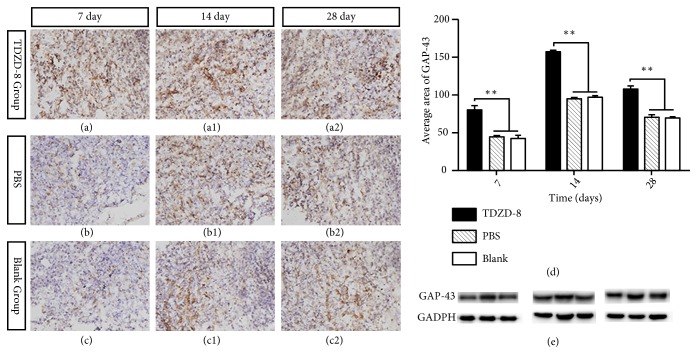
Immunohistochemistry staining for GAP-43 at different time points (a-c). GAP-43 expression was significantly upregulated in the TDZD-8 group (d). Western blot was performed using spinal cord tissue collected 7 d after injury. GAPDH was used as the internal control (e). *∗∗P<*0.05 compared with the PBS and blank groups.

At 7 d after injection, immunohistochemistry and Western blot for GAP-43 showed that GAP-43 was primarily expressed in white matter in structures shaped such as the tan filaments. At each time point, the area expressing GAP-43 was significantly larger in the TDZD-8 group than in the PBS group and blank group. GAP-43 expression peaked at 14 d after injury and then gradually decreased (*P* < 0.05, Figure 2).

### 3.3. Transmission of an FITC Fluorescence Tracer

**Figure 3:**
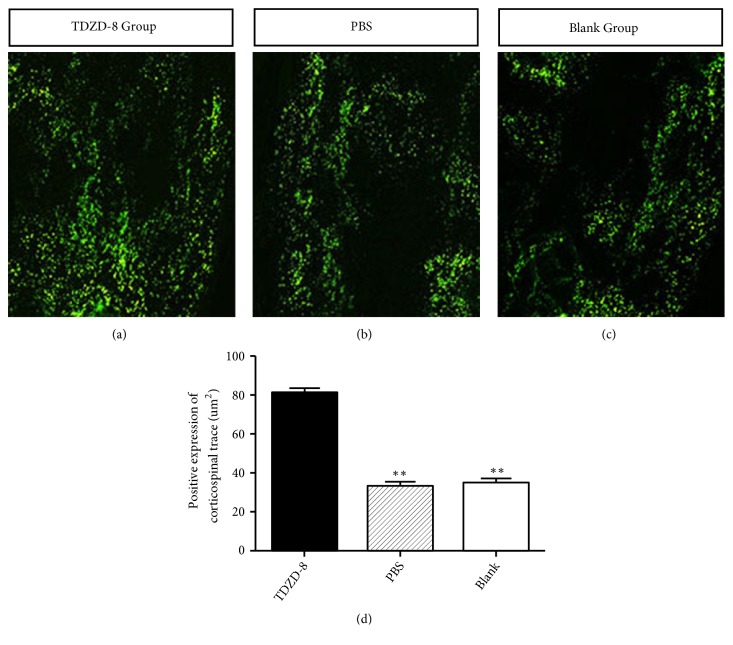
FITC fluorescence traces for observing the progression of nerve fiber regeneration in the trauma area (a-c). At 8 weeks after SCI, more positively labeled cortical spinal tract fibers were observed in the TDZD-8 group (d). *∗∗P<*0.05 compared with the PBS and blank groups.

The FITC tracer was observed to pass through the brain sensorimotor cortex to the SCI area, where structures were obviously disordered and hollowed. In the TDZD-8 group, more positively labeled cortical spinal tract fibers were observed around the injury area, where they presented as short clumps that went in different directions and had different structural disorders. The fluorescence tracer passed through the hollow region caudally. However, in the PBS and blank groups, there were few positively labeled cortical spinal tract fibers around the injury area, and they did not reach the hollow caudal region. At 8 w after injection, the graphic analysis showed that the size of the area containing positively labeled corticospinal tract fibers in the TDZD-8 group was 81.9±1.1 *μ*m^2^, which was significantly larger than the same areas in the PBS group (33.7 ±1.3 *μ*m^2^) and the blank group 35.2 ±2.2 *μ*m^2^ (*P* < 0.05, Figure 3).

### 3.4. BBB Scores of the Lower Limbs

**Figure 4:**
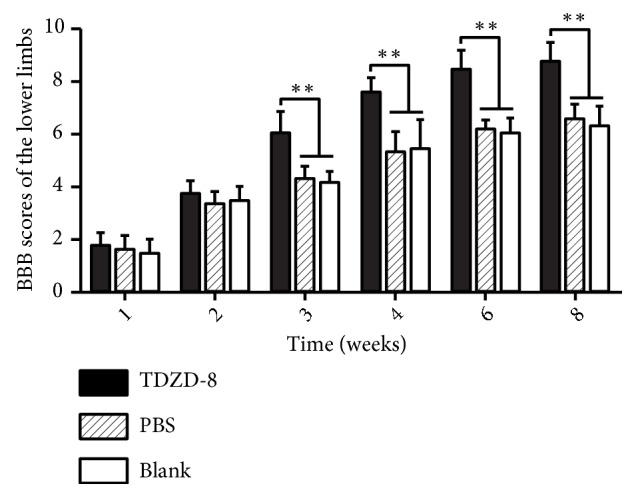
Effect of TDZD-8 on the BBB scores of the lower limbs. The BBB scores of the lower limbs were evaluated in rats at 1, 2, 3, 4, 6, and 8 weeks after SCI. BBB scores were not significantly different among the three groups during the first 2 weeks after SCI (*P* > 0.05) but were significantly higher in the TDZD-8 group beginning at 3 weeks after SCI. *∗∗P<*0.05 compared with the PBS and blank groups.

BBB [10] scores increased after injection in each group. There were no differences among the groups at 1 w and 2 w after injection, but at 3 w after injection, BBB scores were significantly higher in the TDZD-8 group than in the PBS and blank groups (*P* < 0.05, Figure 4).

## 4. Discussion

After SCI, a cystic cavity forms and becomes surrounded by glial scarring; these scars primarily comprise astrocytes, microglia and dense extracellular matrix proteins. Inhibitory factors in glial scars are present on the cell surfaces in the myelin sheath as well as on the surface of extracellular matrix cells. These factors form a physical barrier to axonal regeneration. Previous studies [11, 12] found that these inhibitory factors mainly consist of CSPG family members, which are hinged by different core proteins and sugar chondroitin sulfate glycosaminoglycans. These factors are expressed at higher levels in the immature central nervous system (CNS) but are obviously decreased in mature neurons. However, their expression is increased after SCI. Zhou et al. [13] found that aggrecan inhibitory molecules in glial scars activated integrin kinase (ILK) via GSK-3 signaling to prevent axon regeneration after SCI. Activated ILK inhibits phosphorylation at 9 serine sites on GSK-3 to activate GSK-3, which then acts on multiple protein substrates to induce signaling cascades [6], inhibit microtubule polymerization, assembly and transport, and affect the dynamic balance between microtubules and microfilaments. Activated GSK-3 also plays a role in axon pinocytosis as a specific adhesion factor and dynamically balances the growth cone, which eventually causes its collapse [5, 6]. In addition, the release of lysophospholipid acid, an axon-exclusive factor, from activated platelets also inhibited axon regeneration normally induced by GSK-3*β* signaling following SCI.

Cell apoptosis is an active death process. A variety of intracellular and extracellular factors may participate in this process. These factors include a series of complex cytokines and biochemical chain reactions. Liu et al. [14] found that apoptosis lasted for more than 3 weeks after SCI and that important pathological and physiological changes are subsequent to secondary SCI. Studies have suggested that apoptosis plays a pivotal role in this secondary damage in animal models and in human tissues by causing progressive degeneration in the spinal cord [15, 16]. Zhang et al. [17] and Ashabi et al. [18] found that many nerve cells died due to apoptosis in response to both nervous system trauma and degenerative disease. It is well known that SCI is a process that involves various factors, including oxygen free radicals, inflammatory mediators and nitric oxide-activated macrophages, which cause nerve cell apoptosis after spinal cord injury [19, 20]. Some studies have indicated that apoptosis in neurons and glial cells plays a role in SCI and that inhibiting neuronal and oligodendroglial apoptosis may be a therapeutic strategy [21, 22]. Some authors [23–26] also noted that factors that promote and inhibit apoptosis coexist in the microenvironment of SCI. In these signaling pathways, activation of Akt enhanced the activity of GSK-3 via PI-3K signaling pathways. Activated GSK-3 downregulated the antiapoptotic factor Bcl-2 and upregulated the proapoptotic factor Bax, thus increasing the occurrence of apoptosis. Therefore, GSK-3*β* activation can induce nerve cell apoptosis but can also mitigate apoptosis levels by restraining proapoptotic activity after SCI. Yeste-Velasco et al. [27] observed that GSK-3*β* inhibitors such as lithium chloride (10 *μ*mol/L) and SB-415286 (20 *μ*mol/L) significantly reduced the expression of mitochondrial apoptosis-inhibiting factors, thereby reducing the occurrence of apoptosis. Cuzzocrea et al. [28] used the GSK-3*β* inhibitor TDZD-8 to treat SCI in rats and observed outcomes using TUNEL staining; the results indicated that rats administered TDZD-8 had fewer apoptotic cells in the injury area than the control group. In our study, we found that there were notably fewer TUNEL-positive cells in the TDZD-8 group than in the PBS and blank groups at each time point (P<0.05, Figure 1). Therefore, TDZD-8 can inhibit neuronal apoptosis after SCI and reduce secondary injury to nerve cells.

Axons can regenerate and restore function following a peripheral nerve injury but cannot regenerate following central injury, in which the damage is accompanied by permanent dysfunction. The failure of axons to regenerate following CNS trauma results from decreased intrinsic properties in the affected neurons [29]. The most predominant losses in intrinsic properties that are related to recovery following SCI are the absence of neurotrophic factors and the presence of inhibitory factors in the environment [30–32]. Reports have demonstrated that several key molecular mechanisms and pathways limit axonal sprouting and regeneration following CNS axonal injury. These include myelin or proteoglycan-dependent inhibitory signaling [3, 33–35]. In clinical settings, most SCIs are contusions, not complete ruptures. Animal experiments have shown that if 10% of the spinal cord long tract is preserved after SCI, partial movement functions can be restored [3]. Therefore, the question of how to promote axonal regeneration became the focus of the present study. GAP-43 is a specific factor that is associated with the development and regeneration of nerve cell membrane acid phospholipid protein. These are primarily located in the axonal growth cone, where they promote axonal regeneration by accelerating growth cone serolemma film expansion [36]. In mature CNS, axonal growth and synaptic plasticity are suppressed, and GAP-43 is expressed at only low levels, whereas it is highly expressed following CNS injury [37]. This difference suggests that it may be involved in the repair process after injury. Therefore, the expression of GAP-43 is an important indicator for evaluating axonal regeneration. Ikegami et al. [38] reported that when a combined treatment consisting of ABC chondroitin enzyme (C-ABC) and neural stem cells (NSPCs) was applied to SCI in rats, immunohistochemical staining showed that GAP-43 expression was high in the trauma center and adjacent areas. Cuzzocrea et al. [28] induced T5-T8 SCI in a rat model and found that serious SCI can result in spinal cord edema, which induces the production of inflammatory mediators in addition to nerve cell apoptosis, restrained axonal growth, and increased hind limb dysfunction. However, application of the GSK-3 inhibitor TDZD-8 significantly decreased nerve cell apoptosis, promoted axon regeneration, and improved the function of the hind limbs. In our study, GAP-43 was expressed within days of SCI and reached a peak at 2 weeks after injury. Its expression was higher in the TDZD-8 group than in the PBS and blank groups, and the difference was statistically significant (*P *< 0.05, Figure 2). We also used a fluorescent tracer to label regenerated nerve fibers. There were more positively marked corticospinal tract fibers around the trauma center and reaching the caudal side of the damage cavity in the TDZD-8 group than in the PBS and blank groups (P<0.05, Figure 3). This observation explains how TDZD-8 improved and extended axonal regeneration after SCI. After treatment, BBB motor scores were higher in each group, with significantly higher scores in the TDZD-8 group than in the PBS and blank groups after 3 weeks (P < 0.05, Figure 4). These results show that TDZD-8 promoted functional recovery after SCI in rat lower limbs.

In conclusion, we demonstrated that TDZD-8 inhibited neuronal apoptosis, upregulated GAP-43 expression, increased the density of cortical spinal tract fibers around the injury area, and increased BBB scores in the lower limbs.

## Data Availability

The data used to support the findings of this study are available from the corresponding author upon request.
